# Using the Red Cross wound classification to predict treatment needs in children with conflict-related limb injuries: a retrospective database study

**DOI:** 10.1186/s13017-020-00333-0

**Published:** 2020-09-18

**Authors:** Lisanne van Gennip, Frederike J. C. Haverkamp, Måns Muhrbeck, Andreas Wladis, Edward C. T. H. Tan

**Affiliations:** 1 Department of Surgery, Radboudumc, P.O. Box 9101, 6500 HB Nijmegen, the Netherlands; 2 Division of Surgery, Department of Biomedical and Clinical Sciences, Linköping University, Linköping, Sweden; 3 Center for Disaster Medicine and Traumatology, Linköping University, Linköping, Sweden; 4 Department of Emergency Medicine, Radboudumc, Nijmegen, the Netherlands

**Keywords:** Global health, Pediatric surgery, Wound classification, Extremity injury

## Abstract

**Background:**

The International Committee of the Red Cross (ICRC) implemented the Red Cross wound classification (RCWC) to quickly assess the severity of a wound in conflict settings. A subdivision into wound grades derived from the RCWC consists of grades 1, 2, and 3, and represents low, major, and massive energy transfer, respectively, to the injured tissue. The aim of this observational study is to assess whether the Red Cross wound grade of a pediatric patient’s wound correlates with patient outcomes.

**Methods:**

All pediatric patients (age < 15 years) treated in an ICRC hospital between 1988 and 2014 for conflict-related penetrating extremity injuries were retroactively included. Correlations were assessed between wound grades and number of surgeries, blood transfusions, days hospitalized, and mortality. Stratification analyses were performed to evaluate potential effect modifiers.

**Results:**

The study included 2463 pediatric patients. Pediatric patients with a higher wound grade received significantly more surgeries (grade 1 median 2; grade 3 median 3), more blood transfusions (grades 1 and 3 received 33.9 and 72.2 units per 100 patients, respectively), and were hospitalized longer (grade 1 median 15; grade 3 median 40 days). Mortality rates did not significantly differ. Stratification analyses did not reveal effect modifiers for the association between wound grades and patient outcomes.

**Conclusion:**

The Red Cross wound grade of a pediatric patient’s extremity wound correlates independently with treatment needs. This simple wound grading system could support clinical decision-making and should be integrated into the clinical assessment of weapon-wounded pediatric patients in conflict settings.

## Introduction

Managing conflict-related penetrating injuries can be challenging. These injuries differ from non-conflict-related injuries in mechanism, degree of contamination, variability in tissue damage, patient’s pre-hospital transfer time, and availability of resources in these environments [1, 2]. Performing an adequate wound assessment is therefore crucial. Several classification systems exist to describe wounds and guide its management, such as the Gustilo-Anderson (GA) classification [3], Tscherne classification [4], and the Arbeitsgemeinschaft Osteosynthesefragen (AO) soft tissue grading system [5]. These classifications correlate with patient outcomes including healing and infection rates, need for secondary surgeries and amputation, length of hospitalization, and lifestyle changes [6–10]. However, the Gustilo-Anderson, Tscherne, and AO soft tissue classifications might not suffice for appropriate description of conflict-related weapon wounds. First of all, they might not be sufficiently distinctive, as conflict-related injuries are most often high-velocity injuries and will frequently be considered a grade 3 in the Gustilo-Anderson classification. Additionally, the classifications apply only to injuries related to a fracture.

Therefore, the International Committee of the Red Cross (ICRC) implemented the Red Cross wound classification (RCWC) to navigate wound assessment in conflict areas. The ICRC is a neutral, independent organization that carries out humanitarian initiatives in many ways. It is one of the world’s main organizations providing medical care in conflict areas for weapon-wounded patients. In the 1990s, the RCWC was developed by Dr. R.M. Coupland, a former ICRC surgeon and a current ICRC medical advisor.

The RCWC describes the wound size and the presence or absence of a cavity, fracture, injury to a vital structure, or metallic foreign body (see Table 1). A subsequent classification system derived from the RCWC is the wound grading system (see Table 2), which subdivides wounds into grades 1, 2, or 3 based on wound size, fracture type (if any), and the presence or absence of a cavity. These grades represent the amount of tissue damage and degree of kinetic energy transferred from the projectile to the body tissue [2, 12–14]. The RCWC was intended to systematically assess wound severity and the type of tissue involved in a quick and easy manner; it recognizes wounds as surgical lesions rather than as weaponry phenomena [2, 11, 13, 15]. Its application could be used to audit surgical performance, to establish a scientific approach to war surgery, and to derive information from the field on wound ballistics [13].

**Table 1:** Red Cross wound classification [11]

Wound feature	Definition
E (entry)	Estimate the maximum diameter of the entry wound in cm
X (exit)	Estimate the maximum diameter of the exit wound in cm (X = 0 if no exit)
C (cavity)	Can the “cavity” of the wound take two fingers (finger width) before surgery? **C = 0**, no **C = 1**, yes
F (fracture)	**F = 0**, no fracture **F = 1**, simple fracture, hole, or insignificant comminution **F = 2**, clinically significant comminution
V (vital structure)	Is the brain, viscera, or major vessels injured? **V = 0**, no vital structure injured **V = N** (neurological), penetration of the dura of the brain or spinal cord (includes penetrating injuries of the head or paraplegia due to projectiles) **V = T** (thorax or trachea), penetration of the pleura or of the trachea in the neck **V = A** (abdomen), penetration of the peritoneum **V = H** (hemorrhage), injury of a major peripheral blood vessel, down to the brachial artery in the arm or the popliteal in the leg
M (metallic body)	Bullet or fragments visible on X ray? **M = 0**, none **M = 1**, one metallic body **M = 2**, multiple metallic bodies

V = N, T, and A are subcategories of central wounds; V = H is a subcategory of wounds of the limbs

**Table 2:** Wound grades derived from the Red Cross wound classification [11]

	Skin defect^a^	Cavity	Fracture
Grade 1	< 10 cm AND	Absent AND	Absent or simple fracture
Grade 2	< 10 cm AND	Present OR	With clinically significant comminution
Grade 3	≥ 10 cm AND	Present OR	With clinically significant comminution

^a^Skin defect: size of entry and exit wound combined

For many years, ICRC surgeons have routinely used the RCWC during assessment of patients treated at ICRC-supported hospitals. A significant part of this patient population comprises pediatric patients with serious conflict-related injuries [16–21]. The application of the RCWC has never been studied in pediatric patients specifically. Wounds with a similar Red Cross wound grade are expected to have a different, more severe impact on the pediatric patient in comparison to an adult patient due to differences in physiology and anatomy [22–25]. Moreover, additional guidance in the management of pediatric patients has been requested by healthcare professionals working in conflict zones [26–29]. For those who are less familiar with conflict-related penetrating injuries, clear and robust guidance on wound management is essential. This assistance might be provided by the Red Cross wound grading system. Therefore, the aim of this study is to assess whether the Red Cross wound grade of a pediatric patient’s wound correlates with patient outcomes.

## Methods

A retrospective observational study was performed using an ICRC database, which contained data of 38,312 patients from various time periods between 1988 and 2014. Patients were treated in one of the ICRC-supported field hospitals at the following locations: Goma, Democratic Republic of the Congo; Kabul, Afghanistan; Khao-I-Dang, Thailand; Lokichogio, Kenya; Kandahar, Afghanistan; Novye Atagui, Russian Federation; Peshawar, Pakistan; and Quetta, Pakistan (Table 3). All patients at each hospital were included during the given time periods. The data were originally registered on handwritten patient files based on patient assessment by an attending surgeon. The patient files were manually converted into an anonymous electronic database using Microsoft Office Excel®.

**Table 3:** Specifications per hospital

Hospital location	Period of data collection	Hospital opening date and closing date
Kao-I-Dang, Thailand	Jan 1988–Sept 1992	1979–1993
Lopiding, Lokichogio, Kenya	Mar 1988–Mar 2006	1987–2006
Kabul, Afghanistan	Mar 1990–Jun 1992	1989–1992
Quetta, Pakistan	Apr 1990–Aug 1996	1983–1996
Peshawar, Pakistan	Jun 1990–Apr 1993	1981–1993
	Feb 2009–May 2012	2009–2014
Mirwais, Kandahar, Afghanistan	May 1996–Jun 1999	1996–still open
Novye Atagui, Russian Federation	Sept 1996–Nov 1996	Sept 1996–Dec 1996
Goma, Kivu, Democratic Republic of the Congo	Nov 2012–Oct 2014	2012–still open

**Fig. 1:**
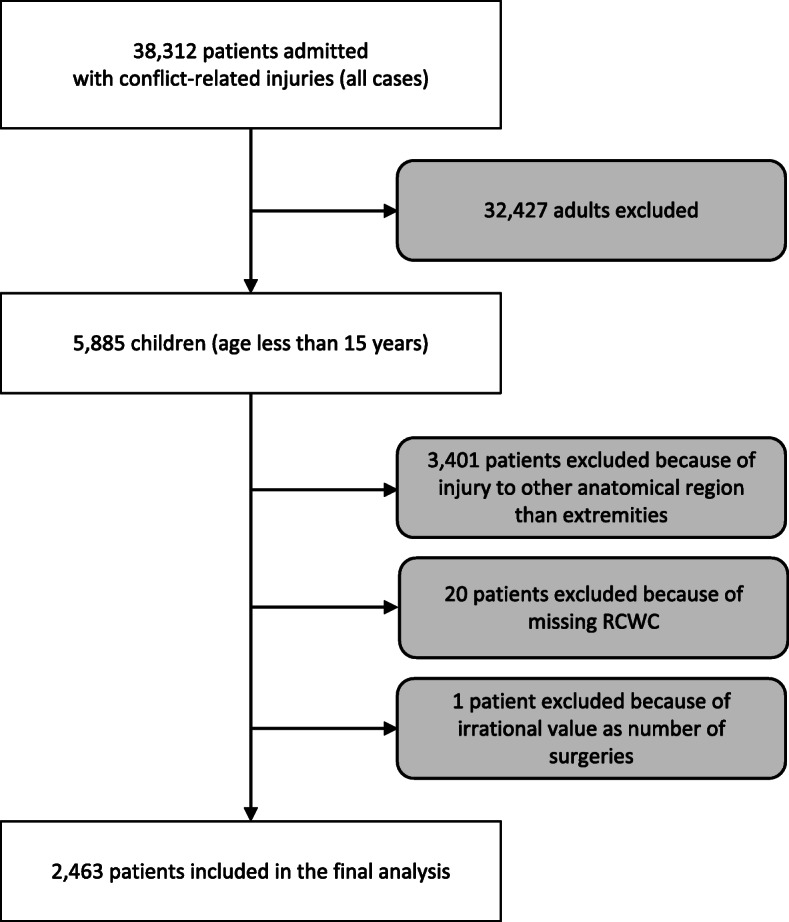
Flow chart of inclusion process for pediatric patients with conflict-related extremity injuries

This study included pediatric patients under 15 years old with conflict-related extremity wounds (Fig. 1). Pediatric patients with wounds to the head, neck, thorax, abdomen, pelvis, buttocks, back, and junctional areas (e.g., axilla or groin) were excluded, because the exact anatomical site in these injuries may define the outcome of the patient to a greater extent than the wound grade. For example, a small wound to the thorax can still be lethal when involving the heart. Furthermore, patients who lacked variables of the RCWC were excluded. Analyses were limited to patients who had the complete data required for that analysis.

Independent variables available in the database were age, gender, mechanism of injury, distribution of limb injuries (upper or lower), time to reach the hospital since sustaining injury, and the RCWC. The available dependent (outcome) variables were number of surgeries, number of blood transfusions, length of hospital stay, and mortality rate. The variable on mechanism of injury is subdivided into gunshot wounds, mine injuries, burn injuries, fragment injuries, and other injuries. Fragment injuries comprise penetration injuries from shells, bombs, or rockets [13]. The following factors were considered possible effect modifiers on the potential correlation between wound grades and patient outcomes: gender, age category (child or adult), time to reach the hospital since sustaining injury, mechanism of injury, and the presence or absence of a fracture. This latter hypothesis was based on clinical reasoning and supported by previous studies that have demonstrated a correlation between these patient factors and outcome or injury severity [8, 21, 30].

All statistical analyses were executed using SPSS statistical software (IBM SPSS Statistics for Windows, Version 25.0). Descriptive analyses were performed for all variables, and results are presented as percentages or median with interquartile range. Comparative analyses were performed between wound grades and among baseline characteristics as well as outcome variables. If patients had multiple wounds registered, the wound with the highest grade was used for comparative analyses between wound grades. A Kruskal-Wallis test was used to determine significant differences for continuous variables, and a chi-square test was used for comparisons of categorical variables. A Bonferroni correction was used for multiple testing. Age, number of surgeries, and length of hospital stay were analyzed as continuous variables. Gender, mechanism of injury, distribution of upper and lower limb injuries, time to reach the hospital since sustaining injury, the RCWC, wound grades, and mortality rate were analyzed as categorical variables in the way they were registered at the initial phase of data entry. The predictive ability was evaluated using *p* values. A two tailed *p* value < 0.05 was considered significant.

Stratification analyses were performed to evaluate the influence of potential effect modifiers on associations between the wound grades and outcome variables (number of surgeries, number of blood transfusions, and length of hospital stay). The outcome variable of mortality was not included in the latter analysis, because of the low absolute numbers of deaths.

Data collected at Peshawar (during 2009–2012) and Goma contained some additional variables which were not registered at the other study locations. These variables concerned patients’ characteristics pre-hospital, on arrival, and in-hospital. Additional descriptive subanalyses were performed with these variables among pediatric patients treated in Peshawar and in Goma.

## Results

The total database (adults included) contained 38,312 patients, of whom 5885 (15.4%) were children under 15 years. The number of pediatric patients who met the inclusion criteria was 2463 (Fig. 1). Regarding the highest wound grade per patient, most pediatric patients had wounds categorized as grade 1 (79.4%, 1 956/2463); less often, patients’ wounds were graded as 2 or 3 (13.9%, 342/2463, and 6.7%, 165/2463, respectively).

Table 4 provides an overview of the study population’s characteristics. The age distribution was approximately the same among all three wound grades with a median of 10 years. There were no significant differences between the three wound grades considering gender distribution (*p* = 0.191, df = 2), with the majority being male: approximately 75% in each category. The number of anatomical sites injured per patient (median 1, IQR 1–2, *p* = 0.441, df = 2) also did not differ significantly among wound grades.

**Table 4:** Subject and injury characteristics per wound grade

	Wound grade 1	Wound grade 2	Wound grade 3	Total
Total number of pediatric patients (%)	1 956 (79.4%)	342 (13.9%)	165 (6.7%)	2463 (100%)
Median age, years (IQR)	10 (7–12)	10 (6.8–12)	10 (8–13)	10 (7–12)
Gender, *N* (%)^a^				
Male	1508 (77.1%)	276 (80.7%)	122 (73.9%)	1 906 (77.4%)
Female	445 (22.8%)	66 (19.3%)	43 (26.1%)	554 (22.5%)
Missing	3 (0.2%)	0 (0.0%)	0 (0.0%)	3 (0.1%)
Mechanism of injury, *N* (%)^a^				
Gunshot wound	517 (26.4%)^§^	195 (57.0%)^#^	90 (54.5%)^#^	802 (32.6%)
Mine injury	474 (24.2%)^#^	61 (17.8%)	29 (17.6%)	564 (22.9%)
Burn	43 (2.2%)^#^	0 (0.0%)	0 (0.0%)	43 (1.8%)
Fragment injury	687 (35.1%)^#^	73 (21.3%)^§^	41 (24.8%)	801 (32.5%)
Other	187 (9.6%)^#^	2 (0.6%)^§^	4 (2.4%)	193 (7.8%)
Missing	48 (2.5%)	11 (3.2%)	1 (0.6%)	60 (2.4%)
Anatomic region of injury, *N* (%)^a^				
Upper limbs (left and/or right)	887 (45.3%)	145 (42.4%)	77 (46.7%)	1 109 (45.0%)
Lower limbs (left and/or right)	1 332 (68.1%)	240 (70.2%)	107 (64.8%)	1 679 (68.2%)
Missing	0 (0.0%)	0 (0.0%)	0 (0.0%)	0 (0.0%)
Fracture, *N* (%)^a^				
Absent	1 785 (91.3%)^#^	87 (25.4%)	31 (18.8%)	1 903 (77.3%)
Present	167 (8.5%)^§^	253 (73.0%)	131 (79.4%)	551 (22.4%)
Missing	4 (0.2%)	2 (0.6%)	3 (1.8%)	9 (0.4%)
Median time since injury, *N* (%)^a^				
< 6 h	480 (24.5%)^#^	70 (20.5%)	22 (13.3%)^§^	572 (23.2%)
6–23 h	417 (21.3%)	60 (17.5%)	36 (21.8%)	513 (20.8%)
24–71 h	276 (14.1%)	66 (19.3%)	22 (13.3%)	364 (14.8%)
72 h or more	723 (37.0%)	140 (40.9%)	81 (49.1%)^#^	944 (38.3%)
Missing	60 (3.1%)	6 (1.8%)	4 (2.4%)	70 (2.8%)

*IQR* interquartile range

^a^Percentages calculated within the wound grades

^#^Statistically significant higher percentage when compared to other wound grades within this variable category (*p* < 0.05)

^§^Statistically significant lower percentage when compared to other wound grades within this variable category (*p* < 0.05)

The time it took for patients to reach the hospital after sustaining the injury was significantly longer in patients with wound grade 3 (*p* = 0.004, df = 6); it took almost 50% (81/165) of these patients 72 h or more to reach the ICRC field hospital. A subsequent analysis comparing the time it took to reach the hospital for different age groups (0–2 years, 3–5 years, 6–9 years, and 10–14 years) revealed that it took pediatric patients of 0–2 years significantly more often over 3 days to reach the hospital (54.5%, 116/213; *p* = 0.000, df = 9). There were no significant differences between gender in the time it took pediatric patients to reach the hospital (*p* = 0.050, df = 3).

Data on patient outcomes per wound grade are listed in Table 5. When a pediatric patient’s wound was graded higher, significantly more surgeries and a longer duration of hospitalization were required (both *p* = 0.000, df = 2). Patients with wound grade 3 required the most blood transfusions with 72.2 units per 100 patients and differed significantly (*p* = 0.000, df = 2) from wound grades 1 and 2 (33.9 and 37.4 units per 100 patients, respectively). Patients with wound grades 1 and 2 did not differ significantly from each other (*p* = 0.266, df = 2). Mortality rates did not differ significantly among wound grades (*p* = 0.091, df = 2).

**Table 5:** Overview of patient outcomes

	Wound grade 1	Wound grade 2	Wound grade 3	Total
Median number of surgeries (IQR)	2 (1–3)^#^	2 (2–3)^#^	3 (2–5)^#^	2 (1–3)
Number of blood products/100 patients	33.9	37.4	72.7^#^	37.0
Median LOS, days (IQR)	15 (7–33)^#^	30 (13–52)^#^	40 (24–68)^#^	18 (8–39)
Mortality rate, *N* (%)	20 (1.0%)	0 (0.0%)	3 (1.8%)	23 (0.9%)

*IQR* interquartile range, *LOS* length of stay in hospital

^#^Statistically significant different from the other wound grades (*p* < 0.05)

Stratification analyses did not demonstrate any trend in the effect on associations between wound grades and outcome variables when stratifying by gender, age category (child or adult), time to reach the hospital since sustaining injury, mechanism of injury, and the presence or absence of a fracture.

### Descriptive subanalyses Peshawar (2009–2012) and Goma (2012–2014)

Sixteen pediatric patients were included in these analyses: 6 patients with wound grade 1, 2 patients with wound grade 2, and 8 patients with wound grade 3. Data on patient characteristics as recorded pre-hospital, on arrival, and in-hospital are listed in Table 6. The greater part of data listed in Table 6 was available only from Goma. Although on arrival all pediatric patients had a blood pressure within normal range for their age, the heart rate was elevated in 4 patients, suggesting (impending) shock. All patients’ wounds were classified as contaminated, and 1 patient with a wound grade 3 had foul odor and discharge from the wound at arrival. One patient developed a wound infection during hospital stay. The other patients had no registered complications.

**Table 6:** Sub analyses Peshawar (2009–2012) and Goma (2012–2014)

	Wound grade 1	Wound grade 2	Wound grade 3	Total
Number of pediatric patients (%)	6 (37.5%)	2 (12.5%)	8 (50.0%)	16 (100%)
Type of pre-hospital care received, *N* (%)^§^				
None	3 (75.0%)	1 (50.0%)	1 (25.0%)	5 (50.0%)
First aid	0 (0.0%)	0 (0.0%)	1 (25.0%)	1 (10.0%)
Medical/emergency care	1 (25.0%)	1 (50.0%)	2 (50.0%)	4 (40.0%)
Surgery	0 (0.0%)	0 (0.0%)	0 (0.0%)	0 (0.0%)
Time to surgery, *N* (%)^§^				
0–6 h	0 (0.0%)	0 (0.0%)	0 (0.0%)	0 (0.0%)
7–12 h	0 (0.0%)	1 (50.0%)	1 (25.0%)	2 (20.0%)
13–24 h	3 (75.0%)	1 (50.0%)	1 (25.0%)	5 (50.0%)
1–7 days	1 (25.0%)	0 (0.0%)	2 (50.0%)	3 (30.0%)
Unstable pulse on arrival, *N* (%)^#,§^	1 (25.0%)	1 (50.0%)	3 (75.0%)	5 (50.0%)
Unstable blood pressure on arrival, *N* (%)^#^	0 (0.0%)	0 (0.0%)	0 (0.0%)	0 (0.0%)
Glasgow coma scale^§^				
13–15	4 (66.7%)	2 (100%)	4 (50.0%)	10 (62.5%)
< 13	0 (0.0%)	0 (0.0%)	0 (0.0%)	0 (0.0%)
Unknown	2 (33.3%)	0 (0.0%)	4 (50.0%)	6 (37.5%)
Body temperature on arrival^§^				
35–36.9	3 (75.0%)	1 (50.0%)	1 (25.0%)	5 (50.0%)
37–38	1 (25.0%)	1 (50.0%)	2 (50.0%)	4 (40.0%)
> 38	0 (0.0%)	0 (0.0%)	1 (25.0%)	1 (10.0%)
Wound type^§^				
Clean	0 (0.0%)	0 (0.0%)	0 (0.0%)	0 (0.0%)
Foul odor and discharge	0 (0.0%)	0 (0.0%)	1 (25.0%)	1 (10.0%)
Contaminated	4 (100%)	2 (100%)	3 (75.0%)	9 (90.0%)
In-hospital complications^§^				
None	4 (100%)	2 (100%)	3 (75.0%)	9 (90.0%)
Infection	0 (0.0%)	0 (0.0%)	1 (25.0%)	1 (10.0%)

Note that not all percentages in this table add up to 100%. This indicates the missing values

^#^Heart rate and blood pressure were categorized as within normal range or not, based on the reference values per age category as listed in the Advanced Trauma Life Support (ATLS) manual [25]

^§^Data missing from Peshawar

Data on several performed surgical procedures were available from both Peshawar and Goma. An amputation above the elbow, an amputation below the knee, an external fixator, and a split skin graft were performed in patients with wound grade 3. One patient with wound grade 3 received two split skin grafts. Delayed primary wound closure was performed in all patients, except for 1 patient with wound grade 3. Most patients underwent at least one wound debridement. Six patients with wound grade 3 needed multiple debridement surgeries, up to five per patient. Re-debridement was not required in wound grades 1 and 2. A change of dressing was more frequently performed on patients with a higher wound grade: maximal one time for patients with wound grades 1 and 2, but up to 10 times for a patient with wound grade 3.

## Discussion

This retrospective database study is the first to provide information on the correlation between the Red Cross wound grade of a pediatric patient’s extremity wound and treatment needs. It comprises a wide-ranging study setting with data from multiple conflict areas over a substantial time period. The results indicate that pediatric patients with higher-graded weapon-related extremity wounds generally require more surgeries per patient, more blood transfusions, and a longer period of hospitalization. This correlation exists independently from gender, age category, time to reach the hospital, mechanism of injury, and the presence or absence of a fracture. Descriptive subanalyses of patients treated in Peshawar and Goma revealed a trend of more invasive surgical procedures in patients with a higher wound grade.

A correlation between the wound grade and mortality was not identified, with the study population showing low in-hospital mortality rates. This could be due to natural triage, which causes more severely injured patients to decease in the field before reaching the medical treatment facility. A previous study by Coupland did demonstrate a statistically significant correlation between a higher wound grade (grade 1 versus grade 2) and mortality in patients with conflict-related abdominal wounds with penetration of the peritoneum or organ injury [31]. Again, the extent of this correlation was also limited due to natural triage, as patients with a wound grade 3 did not show any in-hospital mortality.

In contrast with pre-hospital selection of more stable patients, delay in patient arrival could have increased patients’ treatment needs as their condition has worsened over time. A longer time since injury might lead to presentation with a higher wound grade, since soft tissue damage is often progressive due to microvascular perfusion failure and inflammatory response [32, 33]. No pre-hospital data was available in this study to assess the effect and extent of a possible patient delay. The way this affects the predictability of the RCWC should be subject for future studies.

Remarkable in this study was that each wound grade category consisted of 3 times more boys than girls, while gender distribution of the total population was nearly equal in the countries studied [34]. The unequal in-hospital gender distribution has already been demonstrated by our previous study on pediatric casualties in conflict zones [21] and in other literature [19, 35, 36]. It is thought to be mostly attributable to cultural aspects that cause fewer women to get injured or to access a hospital.

Literature on the predictive value of other wound scores revealed that a higher AO classification of soft tissue injury correlated with a lower primary healing rate, a greater impairment in lifestyle, and a greater likelihood of a second surgery [6]. Whereas that study did not show a correlation between the Gustilo-Anderson classification and patient outcomes, other studies did [7, 8]. It is noteworthy that comparisons between the predictive value of the Red Cross wound grading system and that of other wound classifications can be difficult or misleading, because other wound classifications are mostly related to underlying fractures and corresponding research was often conducted in a civilian setting [6, 7, 9, 10].

When providing medical assistance in a conflict setting, Eshaya-Chauvin and Coupland recommend that at least a certain minimal quantity of blood units should be available at the hospital based on the encountered mechanisms of injury in the hospital region [37]. Our data add to this, suggesting more units of blood are needed for pediatric patients with a higher wound grade. Thus, when a high wound grade is determined during primary assessment, it can support the decision to start preparing blood products or to transport them to the resuscitation room.

The authors realize that the RCWC was initially designed as a descriptive tool and not as a clinical triage tool. Including vital parameters into the RCWC could make it a more suitable tool for triage, which has been previously discussed by Bowyer et al. and Coupland [15, 38]. However, the RCWC should remain easily applicable and vital parameters are already inherently considered during clinical assessment. The authors fully agree with Bowyer et al. that the process of scoring the wound might be equally as important as the classification resulting from it, because the scoring process ensures that important features of the wound are systematically assessed [15].

This retrospective cohort study has its limitations. First, it can be challenging for surgeons to collect data during deployment due to the austere working environment, which may reflect on the accuracy and completeness of the data. The handwritten patient files had to be manually converted into an electronic database, which is an error-prone process. Second, subcategories of wound severity (i.e., the wound grades) were based on three features: wound size (less or more than 10 cm), cavity existence, and fracture type (if any). It is possible that this might not be the most appropriate way of categorizing wound severity, as there could be other cut-off values or features which correlate more strongly with treatment needs than was found in this study. Nevertheless, the wound grading system is a previously acknowledged classification and was therefore retained. Third, the results are applicable only to pediatric patients with isolated extremity wounds, because patients were excluded if they sustained wounds at other anatomical sites. Last, the type of care at the ICRC-supported hospitals has evolved over time and could differ between hospitals or even between surgeons, which could partly account for differences in patient outcomes. Nevertheless, the influence of time and hospital differences on our study results might be diminished by the large amount of data included in this research, originating from multiple hospitals and time periods.

Future research on the RCWC and wound grading is recommended to identify whether a correlation exists with additional patient outcomes, such as the amputation rate and short- or long-term functional impairment. Validity and reliability of the RCWC could be further assessed by calculating the interobserver variability.

## Conclusions

The Red Cross wound grade of a pediatric patient’s extremity wound independently correlates with the required number of surgeries, blood transfusions, and hospitalization time. The application of this easy-to-use grading system ensures systematic evaluation of the wound even in challenging environments, and it could guide clinical decision-making. Healthcare providers in conflict settings should therefore consider implementing the Red Cross wound grade as an essential adjunct in the initial clinical assessment of children.

## Data Availability

The datasets used and/or analyzed during the current study are available from the corresponding author on reasonable request.

## Abbreviations

AO: Arbeitsgemeinschaft Osteosynthesefragen
ICRC: International Committee of the Red Cross
IQR: Interquartile range
RCWC: Red Cross wound classification
